# Digital capability as a multilevel construct: a systematic literature review of its dimensions, antecedents, and consequences

**DOI:** 10.3389/fpsyg.2026.1859025

**Published:** 2026-08-26

**Authors:** Sunu Widianto, Elisabeth Supriharyanti, Zubair Ahmed Pirzada, Nuttawuth Muenjohn, Devi Alviani

**Affiliations:** 1 Faculty of Economics and Business, Universitas Padjadjaran, Bandung, Indonesia; 2 Universitas Katolik Widya Mandala Surabaya, Surabaya, Indonesia; 3 Shah Abdul Latif University, Khairpur, Pakistan; 4 Murdoch University, Murdoch, WA, Australia

**Keywords:** digital capability, digital leadership, digital organization, dynamic capability, systematic review

## Abstract

**Introduction:**

Digital capability (DC) has emerged as a critical construct for understanding how individuals and organizations respond to digital transformation. Despite its growing importance, the literature remains fragmented, with inconsistent definitions, overlapping terminology, and varying levels of analysis across disciplines. This study addresses these issues by synthesizing prior research and positioning DC as a multilevel construct that integrates individual competencies and organizational capabilities.

**Methods:**

This study employs a Systematic Literature Review (SLR) approach, following the guidelines. Articles were retrieved from the Scopus database, covering the period from 2007 to 2022. From an initial pool of 561 studies, 53 peer-reviewed journal articles in the fields of business and management were selected based on predefined inclusion and exclusion criteria. A directed conceptual coding approach was used to identify and classify the antecedents and consequences of DC.

**Results:**

The findings indicate that DC remains conceptually underdeveloped and is predominantly examined at the individual level, particularly in relation to digital skills. At the organizational level, the literature emphasizes dimensions such as digital leadership, digital culture, and digital technology capability. The review identifies key antecedents operating across individual, organizational, and environmental levels, while the consequences of DC are associated with a range of operational and organizational outcomes, including innovation, performance, and resilience. However, empirical evidence on multilevel interactions remains limited, and methodological approaches are largely dominated by quantitative designs.

**Discussion:**

This study contributes to the literature by proposing an integrative, multilevel framework of DC that links its core dimensions, antecedents, and consequences. The findings highlight that DC should be understood as a configurational and context-dependent capability rather than a singular construct. The review also underscores the need for greater conceptual clarity, multilevel empirical research, and broader contextual exploration, particularly in SMEs and developing economies. These insights provide a foundation for future theoretical development and empirical investigation in the field of digital capability.

## 1 Introduction

Most of sectors including healthcare, communication, business, and public services have been greatly affected by digitalization (Farajpour et al., 2022). Digital transformation is being driven more and more by technologies including cloud computing, artificial intelligence, big data, and IoT (Khin and Ho, 2019). Businesses must have digital capabilities (DC) to make use of these tools and improve operations and customer interaction. Lacking them, they run the danger of losing competitiveness (Fitzgerald et al., 2014). Beyond technological adoption, however, digital capability is fundamentally connected to how individuals and organizations learn, adapt, process information, and respond to changing environments. From a psychological perspective, the development of digital capability involves cognitive, motivational, and behavioral processes that enable employees and organizations to acquire digital knowledge, build confidence in using technologies, and translate technological resources into effective action. Consequently, digital capability is not only a technological phenomenon but also a socio-psychological capability shaped by learning, adaptation, leadership influence, and collective organizational behaviors.

Many prior systematic reviewed literatures define digital capability as different and sometimes competing constructs that vary according to disciplinary perspectives and levels of analysis. First stream studies emphasize the technological and infrastructural side of digital capability, highlighting digital platforms, ecosystem connectivity, analytics, IoT, cloud computing, and related technological resources (La Calle et al., 2020; Edu et al., 2020; de Vasconcellos et al., 2021; Heredia et al., 2022). Secondly, previous studies define digital capability more strategically, as an organization’s ability to deploy digital technologies to create value, improve decision-making, and maintain competitiveness (Khin and Ho, 2019; Gao et al., 2022; Wang et al., 2022). A third stream interprets digital capability through the lens of dynamic capabilities, linking it to sensing, seizing, and reconfiguring in digital environments (Annarelli et al., 2021). Despite their shared focus on digital transformation, these perspectives reflect fundamentally different assumptions about the nature of digital capabilities. Some scholars conceptualize them as technological resources, others as organizational competencies, while still others view them as higher-order dynamic capabilities. This conceptual diversity has given rise to unresolved debates about whether digital capabilities should be understood as a set of technological assets, a collection of employee competencies, an organizational capability, or a multilevel construct that simultaneously encompasses both individual and organizational domains. Consequently, similar terms are often used to describe different phenomena, while different terms are sometimes used to describe conceptually overlapping capabilities. This conceptual ambiguity has limited the accumulation of theory and hindered the development of an integrated understanding of digital capabilities.

An integrated understanding of digital capabilities is crucial because digital capabilities emerge through the interaction between individual cognition and the organizational context. Individual factors such as learning orientation, self-efficacy, adaptability, digital confidence, and knowledge acquisition influence the development of digital skills, while organizational factors such as leadership, culture, and social support shape how these skills are collectively utilized. Therefore, understanding digital capabilities requires attention to both individual psychological mechanisms and broader organizational processes. The present review defines digital capability as the ability of an organization to acquire, integrate, reconfigure, and utilize digital technologies, human resources, and organizational processes to create value, support innovation, improve performance, and adapt to changes in the digital business environment (Khin and Ho, 2019; Heredia et al., 2022; Wang et al., 2022; Proksch et al., 2024). This synthesized interpretation is broader than purely technical definitions, but also more precise than treating all digitally related constructs as interchangeable.

**Figure 1:**
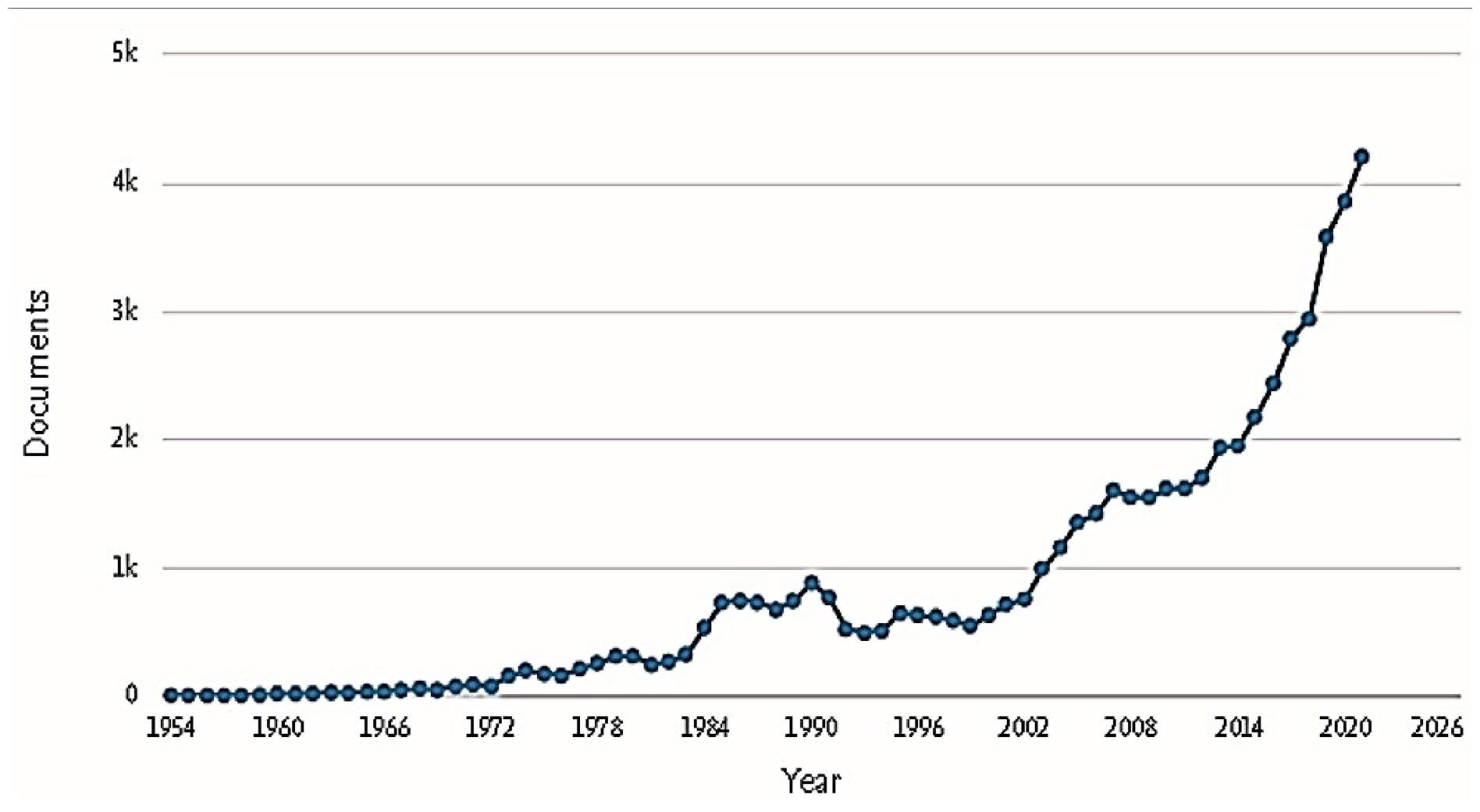
Development of literature on digital capability in Scopus.

Figure 1 shows that research on digital capability (DC) has evolved considerably, beginning with its roots in computer science in the 1950s and expanding rapidly into social science and management disciplines, particularly following major technological advancements and the COVID-19 pandemic. This growth reflects the increasing recognition of digital capability as a critical organizational resource in the digital era.

Both academic and practitioner literature emphasize the importance of developing strong DC within organizations (Westerman et al., 2014). Digital capability enables firms to effectively leverage technologies such as the Internet of Things (IoT), artificial intelligence, and cloud computing, leading to improved organizational performance, more efficient internal processes, and reduced operational costs (Rupeika-Apoga et al., 2022). Moreover, DC enhances firms’ ability to generate timely market intelligence, thereby supporting better strategic planning, higher customer satisfaction, and greater profitability (Khin and Ho, 2019; Gao et al., 2022). Despite these benefits, evidence regarding the outcomes of digital capability remains inconclusive. While numerous studies report positive effects on innovation, organizational agility, and firm performance, others suggest that these benefits depend on contextual factors such as organizational readiness, employee acceptance, leadership effectiveness, and resource availability. These mixed findings indicate that the relationship between DC and organizational outcomes is more complex than a simple direct effect and may involve important mediating and moderating mechanisms.

Research has also highlighted several unresolved issues concerning the antecedents and consequences of DC. For example, Son et al. (2021) identified challenges faced by small and medium-sized enterprises (SMEs), including digital capability asymmetry and the risk of exploitation by technologically advanced partners. Such findings point to the need for a deeper understanding of the conditions that shape the development and impact of digital capability. Another notable characteristic of the literature is the lack of conceptual consensus regarding DC. Existing studies conceptualize digital capability in various ways, including digital innovation capability, IT capability, and digital skills (Xu et al., 2019; Torres de Oliveira et al., 2020). Some researchers focus on organizational-level resources and digital infrastructure (Tan et al., 2013), whereas others emphasize the ability of individuals and organizations to integrate digital technologies into business processes. Furthermore, Vial (2019) positions digital capability as a key enabler of broader digital transformation initiatives. This conceptual diversity highlights the need for a comprehensive review to clarify how digital capability is defined, what factors influence its development, and what outcomes it generates across different organizational contexts.

Digital capability research covers several disciplines, including supply chain (Queiroz et al., 2021), strategy (Gao et al., 2022; Rupeika-Apoga et al., 2022), HR, marketing (Elia et al., 2021), and operations (Mourtzis, 2020), but this breadth has not yet been translated into a clearly integrated understanding of the construct. Past research also tends to concentrate on consumer or supply chain settings (Malchenko et al., 2020; Farajpour et al., 2022). Annarelli et al. (2021) describe DC as higher-order capabilities related to digital integration, digital platform, and digital innovation from a micro-foundational perspective. While previous reviews have documented the definitions, dimensions, and applications of digital capabilities, they have paid limited attention to the psychological mechanisms underlying capability development. Specifically, there remains a limited understanding of how individual processes contribute to the emergence of digital capabilities and how these processes interact with organizational-level factors. Consequently, the psychological underpinnings of digital capabilities remain fragmented across various research streams.

This study on digital capability (DC) adopts an integrative perspective by incorporating individual-level elements, such as digital skills, alongside organizational-level dimensions, including leadership, culture, and technological capability, within a unified review framework. Drawing on 53 articles selected from an initial pool of 561 studies through a systematic literature review approach following Tranfield et al. (2003), this study seeks to clarify the conceptualization of digital capability, synthesize its antecedents and consequences as reported in prior research, and establish a more explicit foundation for future theory development. More specifically, the objectives of this study are articulated through the following research questions:

RQ1: What insights does the existing literature provide regarding the current state of digital capability?

RQ2: What antecedents and consequences of digital capability are identified in the extant literature?

RQ3: What potential avenues are available for future scholars to further develop and extend the digital capability literature?

This study makes three main contributions. First, it deepens the understanding of DC by positioning it as an organizational capability rather than as a narrow technological resource or isolated skill set. This is important because organizational capabilities emphasize not only the possession of digital tools, but also the coordinated ability to deploy leadership, culture, human competences, and technological infrastructure in ways that support adaptation, innovation, and value creation (Khin and Ho, 2019). Second, the study provides an integrative synthesis of the literature by clarifying how related constructs such as digital skills, IT capability, and digital innovation connect to, but do not fully replace, the broader concept of DC. Third, the review develops a consolidated agenda for future research by identifying unresolved theoretical, empirical, and methodological gaps, thereby offering guidance to both scholars and practitioners seeking to better understand how DC is developed and how it generates outcomes across contexts.

## 2 Method

This study employed a Systematic Literature Review (SLR) methodology, following the guidelines proposed by Tranfield et al. (2003). This study adopted a structured approach to identifying, evaluating, and synthesizing literature related to digital capabilities (DC). In the management field, systematic reviews generally follow a three-stage process: planning the review, conducting the review, and reporting and disseminating the findings.

### 2.1 Planning stage

The planning stage involves determining the scope of the review and formulating research questions (Alviani et al., 2024). The scope of the review includes digital capability because it is increasingly recognized as a critical factor supporting digital transformation, innovation, and organizational competitive advantage. The research questions were formulated based on knowledge gaps identified in previous studies, particularly regarding the diversity of conceptualizations of digital capability, the dimensions that shape the construct, the factors that influence it, and its impact on organizations. Therefore, this review aims to answer several key questions: (1) how is digital capability conceptualized in the literature; (2) what antecedents and consequences of digital capability have been identified in previous research; and (3) what recommendations can be made for a research agenda to support the development of future digital capability studies. The research questions were answered by developing a review protocol to ensure the research process was conducted systematically and could be replicated. The protocol included determining the databases used, the literature search strategy, inclusion and exclusion criteria, article screening procedures, data extraction, reporting, and data synthesis.

### 2.2 Implementation stage

The implementation stage includes database searches, screening, study selection, and data extraction. During the literature search, screening, and selection phase, this study applied a series of inclusion and exclusion criteria to ensure the quality and relevance of the selected studies. Scopus was chosen as the primary database due to its extensive coverage of high-quality journals in business, management, information systems, and related disciplines (Harzing and Alakangas, 2016; Zhu et al., 2021). The literature search was conducted using keywords related to “digital capabilities” and related terms. The search was conducted in Scopus using a combination of the keywords “digital capabilities,” “digital competence,” “digital skills,” “digital transformation capabilities,” and related terms identified through the preliminary search. The search process was not limited to a specific year; instead, we prioritized published scientific articles because they had undergone a rigorous peer-review process and evaluation, ensuring the research was conducted with high scientific rigor. Scientific articles are considered the most accurate, trustworthy, and reliable sources of information (Anderson et al., 2020; Azarian et al., 2023; Smela et al., 2023). Therefore, we excluded editorials, perspectives, conference proceedings, book chapters, or other contributions that were not peer-reviewed (López-Duarte et al., 2016). Inclusion criteria were limited to business and management publications and publications in English, excluding those in other languages (Follmer and Jones, 2018), both due to the language limitations of research scholars (Battisti et al., 2021) and the perceived limited impact of such contributions on international academic discourse (Gallardo-Gallardo and Thunnissen, 2016). The screening process was conducted in three stages. First, titles and abstracts were reviewed to eliminate clearly irrelevant studies. Second, only articles with available full text were considered (Vrontis and Christofi, 2021). Third, the remaining articles were evaluated for their conceptual relevance to digital capabilities and their ability to contribute to the review question. From the initial pool of 561 articles, several studies were excluded due to: being outside the business and management domain (321 articles), and lacking a focus on digital capabilities or not providing empirical or conceptual contributions (86 articles). The final sample consisted of 53 studies that met all inclusion criteria and were subjected to detailed coding and analysis.

The study selection process, including screening and coding, was conducted by two independent reviewers. During the screening stage, the two reviewers reviewed titles, abstracts, and full texts based on predetermined inclusion and exclusion criteria. Differences in selection decisions were discussed until a consensus was reached. The same procedure was applied to the data coding process, specifically identifying conceptualizations of digital capabilities, construct dimensions, theoretical foundations, antecedents, and consequences. The use of two independent reviewers was implemented to reduce potential subjective bias and increase consistency in the data extraction process. The data extraction process followed the recommendations of Tranfield et al. (2003). Relevant data were extracted and recorded in an Excel spreadsheet, which included details such as author names, year of publication, research objectives, research design and data, theoretical framework, summary of research findings, conceptualization of digital capabilities, construct dimensions, antecedents, and consequences.

### 2.3 Reporting stage

**Figure 2:**
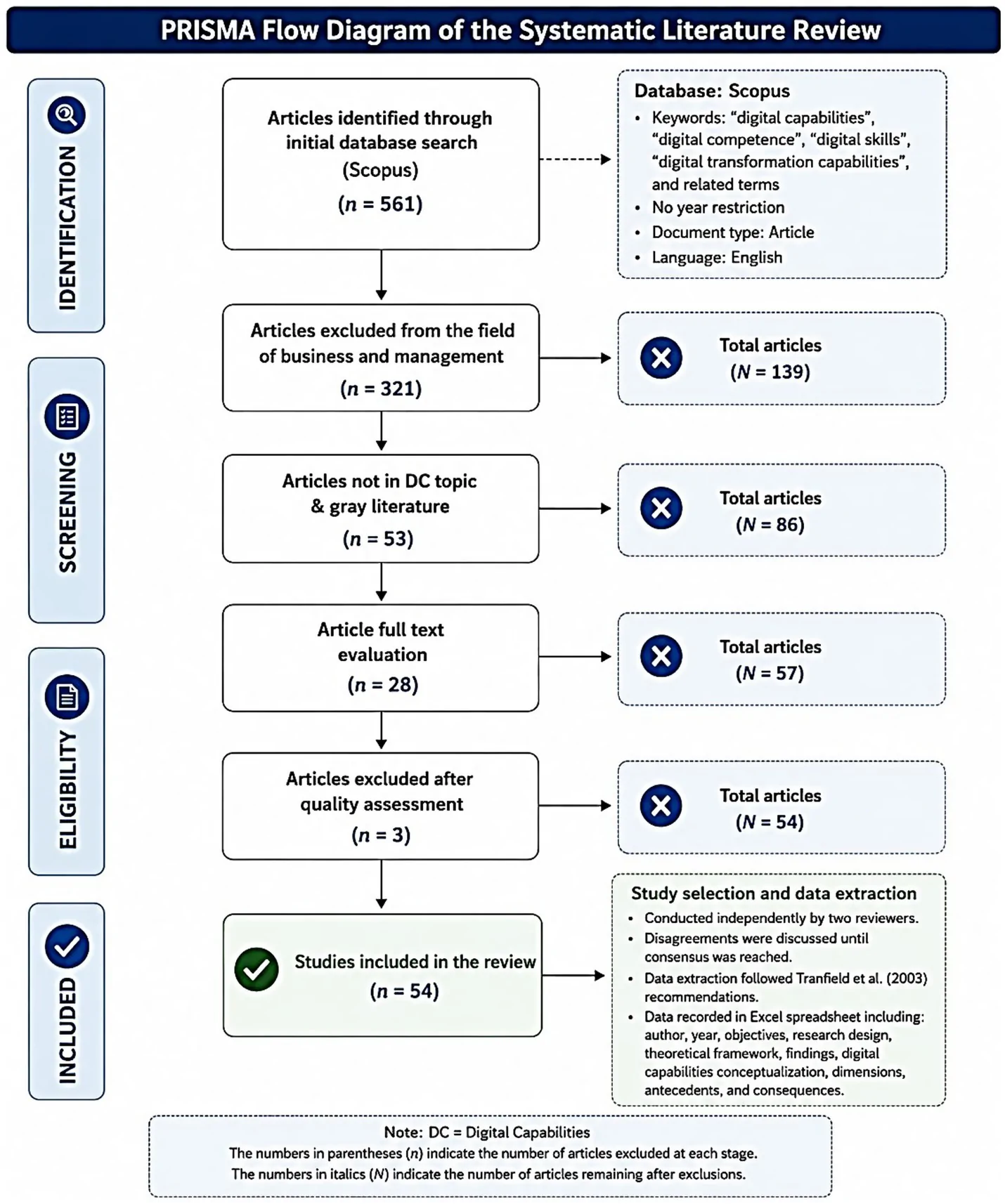
Flow diagram of searched, screened, and included studies.

The reporting stage focuses on synthesizing and presenting findings aligned with the stated objectives of the review. First, to answer RQ1, this review examines the current state of digital capability research, including its definitions, dimensions, and dominant indicators. Particular attention is paid to how digital capability has been conceptualized across studies and how its core dimensions emerge in the literature. Second, to answer RQ2, this review systematically identifies and categorizes the antecedents and consequences of digital capability reported in previous studies. Antecedents are classified as factors that facilitate or shape the development of digital capability, while consequences refer to individual- and organizational-level outcomes associated with digital capability. Third, to answer RQ3, this review synthesizes unresolved conceptual, theoretical, methodological, and empirical gaps identified across the literature. Based on these gaps, future research directions are proposed to advance the understanding of digital capability (Figure 2).

## 3 Results

### 3.1 Current state of digital capability research

This section synthesizes the overall state of digital capability (DC) research by examining publication trends, journal outlets, theoretical foundations, and methodological characteristics. Together, these findings provide an overview of how the DC literature has evolved, how the construct has been conceptualized, and the extent to which the field has matured.

**Figure 3:**
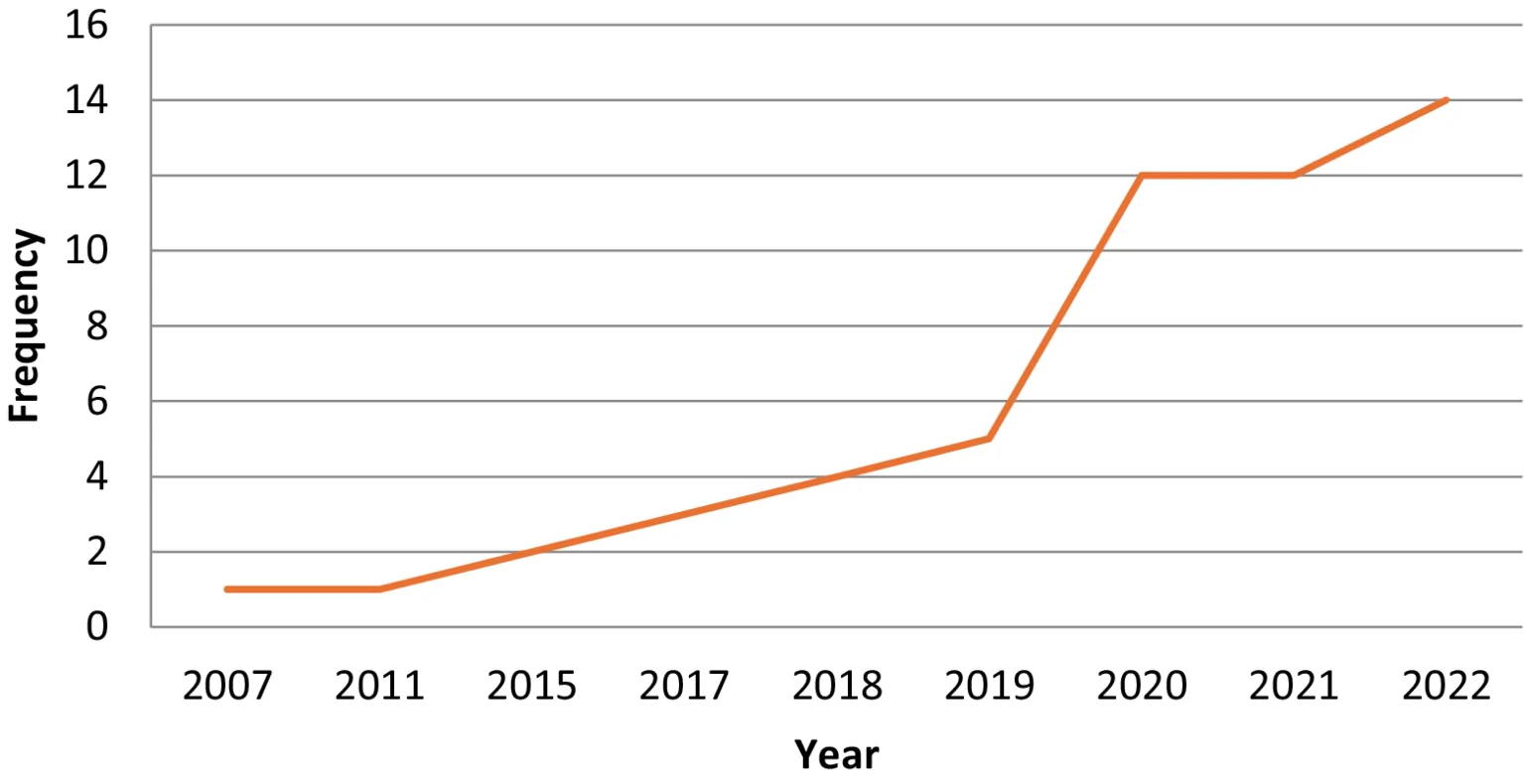
Growth of the reviewed DC literature.

The review identified 53 articles that met the inclusion criteria. The selected studies were published between 2007 and 2022, indicating that DC research is a relatively recent but rapidly expanding field. As shown in Figure 3, publication activity remained limited during the early years but increased steadily after 2011. A notable acceleration occurred after 2019, coinciding with widespread digital transformation initiatives and the organizational disruptions associated with the COVID-19 pandemic. During this period, organizations increasingly relied on digital technologies to maintain operational continuity, stimulate innovation, and support strategic adaptation, resulting in growing scholarly interest in digital capability (Rupeika-Apoga et al., 2022).

The quality of the reviewed studies is reflected in their publication outlets. As presented in Table 1, most articles were published in highly ranked journals. Specifically, 72.20% (39 studies) appeared in Scopus Q1 journals, while the remainder were distributed across Q2, Q3, and Q4 journals. The dominant outlets were located within the fields of strategic management, information systems, innovation management, and operations management. This concentration suggests that digital capability has primarily been examined as a strategic and organizational phenomenon rather than solely as a technological issue. Overall, the publication trend and journal quality indicate that DC has emerged as an increasingly important area of inquiry, particularly in response to accelerating digital transformation across industries. However, despite the growing volume of studies, the literature remains conceptually fragmented, with varying definitions, theoretical perspectives, and levels of analysis.

**Table 1:** List of journal outlets.

Scopus index	Journal name	Frequency
Q1	Asia Pacific Management Review	1
	Benchmarking	1
	European Journal of Innovation Management	1
	IEEE Transactions on Engineering Management	1
	Industrial Marketing Management	1
	Information and Management	1
	Information Systems Research	2
	International Journal of Construction Management	1
	International Journal of Innovation Science	2
	International Journal of Operations and Production Management	1
	International Journal of Production Economics	2
	International Journal of Production Research	2
	International Journal of Retail and Distribution Management	1
	International Marketing Review	1
	Journal of Business Economics	1
	Journal of Business Research	3
	Journal of Cleaner Production	2
	Journal of Enterprising Communities	1
	Journal of Innovation and Knowledge	1
	Journal of Management Development	1
	Journal of Marketing Communications	1
	Journal of Retailing and Consumer Services	1
	Journal of Small Business Management	1
	Journal of Theoretical and Applied Electronic Commerce Research	1
	MIS Quarterly: Management Information Systems	2
	Project Management Journal	1
	Strategic Change	2
	Technological Forecasting and Social Change	1
	Technovation	2
Total		39
Q2	Australasian Journal of Information Systems	1
	Competitiveness Review	1
	International Journal of Information Systems and Project Management	1
	Journal of Business Strategy	1
	Journal of Global Information Management	1
	Journal of Industrial Engineering and Management	1
	Australasian Journal of Information Systems	1
Total		6
Q3	Development and Learning in Organizations	1
	International Journal of Information Systems and Change Management	1
	International Journal of Pharmaceutical and Healthcare Marketing	1
	Revista de Administracao Mackenzie	1
	South African Journal of Business Management	1
Total		5
Q4	Journal Women’s Entrepreneurship and Education	1
	International Journal of Applied Business and Economic Research	1
	Pertanika Journal of Social Sciences and Humanities	1
Total		3

### 3.2 Theoretical lenses applied in digital capability research

Research on digital capability employs a variety of theoretical perspectives to explain how digital capabilities are formed, developed, and influence organizational performance, although not all studies explicitly base their analyses on a particular theory. Of the 53 articles reviewed, the majority adopted the Resource-Based Theory (RBT/RBV) and Dynamic Capability Theory (DCT) as their primary theoretical foundations. This finding indicates that digital capability is generally viewed as a strategic resource and organizational capability that can create sustainable competitive advantage (Barney, 1991; Teece, 2007). Resource-Based Theory (RBT) is the most dominant theoretical perspective in digital capability research. This theory is used to explain that digital capability constitutes a strategic resource that is valuable, rare, difficult to imitate, and non-substitutable, thereby enhancing organizational performance. Studies applying RBT include Mishra et al. (2007), Elia et al. (2021), Sánchez-Montesinos et al. (2018), Galera-Zarco (2024), Wiesböck et al. (2020), Chi et al. (2018), and Rupeika-Apoga et al. (2022). Other studies combine RBT with additional perspectives, such as Dynamic Capability Theory and Ambidexterity Theory, to explain how organizations manage digital resources in dynamic environments. These findings suggest that digital capability is frequently positioned as a strategic asset that enables organizations to achieve competitive advantage and improve business performance (Barney et al., 2011; Alviani et al., 2024). Dynamic Capability Theory (DCT) represents the second most frequently used theoretical perspective. This theory emphasizes an organization’s ability to integrate, build, and reconfigure resources in response to rapid environmental changes driven by digital transformation. Studies employing DCT include Khin and Ho (2019), Oliveira et al. (2023), Witschel et al. (2019), Heredia et al. (2022), Huikkola et al. (2022), and Shamaki et al. (2022). The use of this theory demonstrates that digital capability is not merely understood as the possession of technology but also as an organization’s ability to adapt, innovate, and continuously respond to market changes (Teece et al., 2016; Alviani et al., 2024).

Several studies have also adopted alternative theoretical perspectives to explain specific aspects of digital capability. Social Cognitive Theory was employed by Wei et al. (2011) to explain the digital divide from the perspective of individual behavior and social factors influencing technology use. Complexity Theory was utilized by Park and Mithas (2020) to understand the complexity of digital business strategies within organizations. Meanwhile, Resource Dependence Theory was applied by Mosch et al. (2022) and Son et al. (2021) to explain how resource dependency and imbalances in digital capability affect interorganizational relationships within supply chains. Penrosian Growth Theory was used by Scuotto et al. (2021) to explain firm growth through the development of digital resources and capabilities. Contingency Theory was adopted by Proksch et al. (2024) to highlight the importance of alignment between digital strategy, organizational culture, and digital capability. Leader-Member Exchange (LMX) Theory was employed by Nasution et al. (2020) to understand the role of leader-member relationships in digital competence development. Furthermore, Organizational Information Processing Theory was used by Zhang et al. (2022) to explain how digital capability influences information transparency and the behavior of business partners. Adoption and Diffusion Theory was applied by Seclen-Luna et al. (2022) to understand the dissemination and utilization of digital technologies within organizations. Notably, several reviewed articles did not explicitly specify the theoretical foundations underlying their studies. These studies generally focused on the development of conceptual frameworks, digital maturity assessments, digital transformation case studies, or the identification of digital capability dimensions without linking them to a specific theoretical perspective. This condition indicates that the field of digital capability remains in a developmental stage and has not yet established a dominant theoretical paradigm. Nevertheless, the predominance of Resource-Based Theory and Dynamic Capability Theory suggests that digital capability research generally views digital capability as a source of competitive advantage that must be continuously developed and adapted to dynamic business environments (Teece, 2007; Barney et al., 2011; Alviani et al., 2024).

### 3.3 Methodological profile of the reviewed studies

The analysis of 53 articles reveals that research on digital capability is dominated by quantitative approaches, with approximately two-thirds of the studies employing surveys and statistical analyses to examine the relationships between digital capability, antecedent factors, and various organizational outcomes. Quantitative studies typically involve respondents such as managers, business owners, employees, professionals, and students to assess the impact of digital capability on performance, innovation, digital transformation, and productivity (Khin and Ho, 2019; Heredia et al., 2022; Rupeika-Apoga et al., 2022). We also found that qualitative approaches are widely used, particularly to explore the processes of digital capability development, digital transformation, digital servitization, and business model change. Qualitative studies commonly employ case studies, in-depth interviews, action research, and conceptual analyses, involving company leaders, senior managers, and experts as key informants (Witschel et al., 2019; Huikkola et al., 2022). Several studies have adopted mixed-methods approaches, combining quantitative and qualitative techniques to achieve a more comprehensive understanding of digital capability. This approach is frequently applied in studies focusing on model development, digital maturity assessment, and the evaluation of organizational digital transformation initiatives (Nair, 2019; Nasution et al., 2020; Robertson et al., 2022). Finally, a number of studies employ specific methodologies such as grounded theory, longitudinal studies, and literature reviews to develop conceptual frameworks and models of digital capability development (Adhikari et al., 2017; Queiroz et al., 2019; Devi et al., 2022).

When examined from the perspective of the unit of analysis, the majority of studies focus on organizations. This finding indicates that digital capability is most commonly conceptualized as a strategic capability possessed by firms or organizations to address the challenges of digital transformation. Nevertheless, several studies use individuals as the unit of analysis, particularly in the contexts of the digital divide, digital skills, digital mastery, and work productivity. Some studies even adopt a multilevel perspective by linking individual digital capabilities to organizational outcomes, while others employ broader units of analysis such as industries or countries (Wei et al., 2011; Chang et al., 2015; Scuotto et al., 2021). With regard to respondents, the most frequently represented groups are managers, business owners, organizational leaders, and senior executives, as they are generally considered to possess adequate knowledge of digital strategies and organizational capabilities. In addition, several studies include employees, professionals, academics, students, and entrepreneurs as primary sources of data, depending on the specific research context (Shin H. I. and Kim J., 2017; Wiesböck et al., 2020; Afrianty et al., 2022).

## 4 Discussion

### 4.1 Definitions and conceptualizations of digital capability

Digital capability can be defined as an organization’s ability to acquire, integrate, reconfigure, and utilize digital technologies, human resources, and organizational processes to create value, support innovation, improve performance, and adapt to changes in the digital business environment (Khin and Ho, 2019; Heredia et al., 2022; Wang et al., 2022; Proksch et al., 2024). From a dynamic capabilities perspective, digital capability enables companies to adapt and innovate (Teece, 2018) through the use of intelligent analytics, thereby creating value for the organization (Wang et al., 2022). However, digital capability is formed through individual interactions (Felin et al., 2012; Scuotto et al., 2021), built through a bottom-up process derived from individual contributions (Klein and Kozlowski, 2000; Helfat and Peteraf, 2009). This means that digital transformation initiatives have the potential to fail if they are not supported by adequate employee digital literacy (Hess and Rothaermel, 2011).

Digital capability encompasses not only technological aspects, but also individual digital skills, digital leadership, and organizational culture that enable digital transformation and achieve sustainable competitive advantage (Nasution et al., 2020; Scuotto et al., 2021; Devi et al., 2022; Robertson et al., 2022). Therefore, the DC dimension is broken down into digital skills, digital leadership, digital culture, and digital technology capability. The identification of the four dimensions is the result of an iterative synthesis of the reviewed studies. During the coding process, all constructs used to define, operationalize, explain, or develop digital capability were extracted and compared across studies. The analysis revealed substantial variation in terminology, as follows (Table 2).

**Table 2:** Dimensions of digital capability.

**Specific indicators**	**Indicators**	**Dimensions**	**Authors**
Digital literacy, information literacy, software skills, technology use skills, digital competence, employee digital capability, digital talent, digital orientation, individual digital capability	Individual capability to use digital technologies	Digital skills	Khin and Ho (2019), Queiroz et al. (2021), Scuotto et al. (2021), Devi et al. (2022), Wei et al. (2011), Nasution et al. (2020), Afrianty et al. (2022), Proksch et al. (2024), and Nair (2019)
Strategic vision for digital transformation, digital mastery, management support, leadership commitment, digital governance, orchestration of digital initiatives, alignment strategy, digital transformation leadership	Strategic direction and transformation management	Digital leadership	Nasution et al. (2020); Robertson et al. (2022); Devi et al. (2022); Park and Mithas (2020)
Openness to change, experimentation, innovation climate, collaboration culture, technology-supportive values, organizational readiness, digital mindset, learning orientation	Organizational norms and values supporting digitalization	Digital culture	Proksch et al. (2024) and Khin and Ho (2019)
IT capability, IT infrastructure, analytics capability, IoT capability, cloud capability, platform capability, e-collaboration capability, digital infrastructure, interoperability, digital resources, technological capability	Technological and infrastructural capability	Digital technology capability	Ardolino et al. (2018), Chaudhuri et al. (2022), Elia et al. (2021), Heredia et al. (2022), Hidalgo et al. (2020), Khin and Ho (2019), Seclen-Luna et al. (2022), Wiesböck et al. (2020), Witschel et al. (2019), Proksch et al. (2024), and Nair (2019)

We grouped conceptually similar constructs into higher-level categories. Constructs related to employees’ ability to use, understand, and apply digital technologies were consolidated under the digital skills dimension (Wei et al., 2011; Scuotto et al., 2021; Afrianty et al., 2022). Constructs describing managerial vision, strategic direction, change orchestration, and the ability to mobilize digital transformation initiatives were categorized as digital leadership (Nasution et al., 2020; Park and Mithas, 2020; Robertson et al., 2022). Organizational values, norms, openness to experimentation, and technology-enabling behaviors were grouped under digital culture (Khin and Ho, 2019; Proksch et al., 2024). Finally, constructs referring to digital infrastructure, IT resources, analytics, platforms, interoperability, and technology systems were synthesized into digital technology capabilities (Ardolino et al., 2018; Wiesböck et al., 2020; Heredia et al., 2022). The selection of these four dimensions was guided by two criteria. First, they emerged consistently across a large number of the studies reviewed. Second, they play a central conceptual role in explaining how digital capabilities are developed and realized. Other frequently cited concepts, such as digital orientation, digital maturity, organizational learning, digital innovation, and firm performance, were not retained as core dimensions because the literature largely treats them as antecedents, contextual conditions, mediating mechanisms, or outcomes of digital capabilities, rather than as dimensions of the construct itself.

#### 4.1.1 Digital capabilities as a multilevel construct

**Figure 4:**
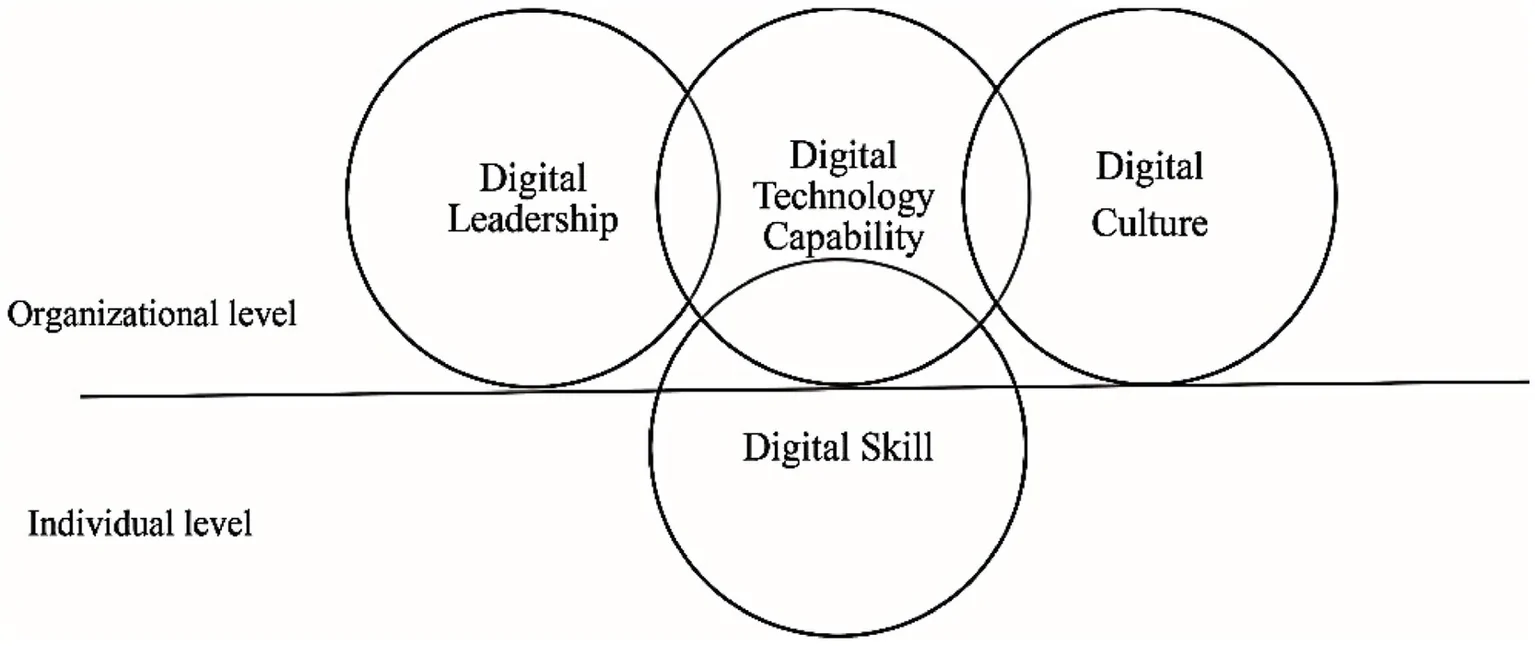
Dimensions of DC.

A key observation emerging from this review is that digital capability encompasses multiple levels of analysis (Figure 4). The studies reviewed collectively indicate that digital capability cannot be adequately explained solely by individual competencies or organizational resources. Rather, the construct appears to emerge from the interaction between employee-level capabilities, managerial actions, organizational context, and technological resources. At the individual level, the literature primarily focuses on digital skills, which refer to the ability to use digital technologies effectively (Wei et al., 2011; Hidalgo et al., 2020; Scuotto et al., 2021; Afrianty et al., 2022). These studies emphasize that organizational digitalization ultimately depends on individuals’ capacity to adopt, interpret, and apply digital technologies in their daily work activities. From a microfoundational perspective, such individual actions constitute the building blocks through which higher-level organizational capabilities are developed. At the organizational level, studies predominantly conceptualize digital capability through digital leadership, digital culture, and digital technology capability (Nasution et al., 2020; Park and Mithas, 2020; Robertson et al., 2022; Proksch et al., 2024). These dimensions reflect the strategic, cultural, and infrastructural conditions that enable organizations to coordinate digital resources and translate employee competencies into collective outcomes. In this regard, organizational-level mechanisms shape the environment in which individual digital capabilities are developed and deployed.

#### 4.1.2 Digital skills

Digital skills are often regarded as a fundamental component in the development of organizational digital capability (DC). In the literature, this concept is also referred to as digital competencies (Hidalgo et al., 2020), digital abilities (Scuotto et al., 2021), or digital talent, reflecting individuals’ capacity to effectively utilize digital technologies in performing tasks and creating value for the organization. According to Devi et al. (2022), the development of organizational capabilities is grounded in individuals’ fundamental characteristics, such as digital mindset, digital literacy, and the ability to create value through technology. Similarly, Scuotto et al. (2021) define digital skills as the capacity to recognize, understand, and utilize information technologies to support the achievement of organizational objectives. These capabilities enable organizations to adopt and leverage new technologies more rapidly and effectively (Kohli and Grover, 2008).

The relationship between individual digital skills and organizational digital capability can be explained through the microfoundations perspective, which emphasizes that organizational capabilities are rooted in the knowledge, skills, and actions of individuals within the organization (Helfat and Peteraf, 2009). From this perspective, digital capability does not emerge automatically at the organizational level; rather, it is built through the accumulation of employees’ digital competencies. However, the findings of this literature review indicate that empirical evidence regarding such multilevel mechanisms remains limited. Most studies tend to focus either on individual-level factors, such as employee skills and competencies, or on organizational-level factors, such as digital leadership and technological resources. Only a small number of studies explicitly examine cross-level interactions simultaneously. Therefore, the multilevel nature of digital capability should currently be viewed as a promising theoretical proposition rather than a fully validated empirical conclusion.

A growing body of research suggests that individual digital skills make a significant contribution to the development of organizational digital capability. These skills are closely associated with digital leadership (Nasution et al., 2020), information technology capability (Afrianty et al., 2022; Proksch et al., 2024), and the effective utilization of technology within organizations. Employees’ knowledge and experience facilitate more optimal use of technology (Bassellier et al., 2001; Arkhipova and Bozzoli, 2018) while also enhancing collaboration between humans and digital technologies (Queiroz et al., 2021). Furthermore, individuals’ digital orientation (defined as the degree of commitment and openness toward the use of digital technologies) has been shown to strengthen organizational digital capability (Afrianty et al., 2022). Over time, the accumulation of digital skills, experience, and digital orientation forms the foundation that enables organizations to adapt and innovate in increasingly digitalized environments (Wei et al., 2011).

The development of digital skills requires systematic organizational support. Khin and Ho (2019) emphasize the importance of competency development programs, incentives, and management practices aligned with digital transformation initiatives. Such support can be realized through continuous training, technology-enabled learning, and reward mechanisms that encourage the adoption of digital technologies (Lewis et al., 2004). In addition, Proksch et al. (2024) demonstrate that employees’ digital skills generate a greater impact when supported by a strong digital culture and robust information technology capabilities. Therefore, digital skills function not only as an individual resource but also as a critical foundation for the development of organizational digital capability, enabling change, innovation, and sustainable competitive advantage.

#### 4.1.3 Digital leadership

Digital leadership represents an organizational-level capability that plays a critical role in guiding organizational transformation and development through the effective use of digital technologies (Nasution et al., 2020). The concept refers to leaders’ ability to integrate business and information technology resources to build, manage, and optimize organizational digital capability (DC) (Park and Mithas, 2020). Leaders play a strategic role in formulating digital vision and strategy, managing organizational change, and developing the digital talent and competencies required to support organizational objectives in the context of digital transformation (Wang and Zeng, 2017; Devi et al., 2022).

The literature suggests that digital leadership functions as a key mechanism linking digital resources to an organization’s ability to adapt and innovate. In environments characterized by uncertainty, leaders play an important role in directing the development of digital skills and creating conditions that support the effective use of technology (Benítez-Núñez et al., 2024). Visionary and adaptive leaders can shape the direction of organizational digital transformation by aligning business and technology strategies, thereby enhancing the effectiveness of digital initiatives (Westerman et al., 2014). Furthermore, because digital technologies can be relatively easily imitated by competitors, sustainable competitive advantage increasingly depends on an organization’s ability to manage data, business processes, and human resource competencies. In this regard, leaders play a crucial role in integrating technology into organizational work processes while simultaneously fostering the development of employees’ digital skills (Blosch and Burton, 2014; Sabau, 2016).

A growing body of empirical research supports the importance of digital leadership in building organizational digital capability. Digital leadership has been shown to enhance organizational growth through the alignment of business strategies and digital technologies, particularly among small and medium-sized enterprises. Moreover, digital leadership contributes to the success of digital transformation by strengthening organizational resilience in response to environmental changes and enhancing the organization’s ability to achieve digital mastery (Nasution et al., 2020; Robertson et al., 2022). These findings indicate that digital leadership functions not only as a driver of digital transformation but also as a strategic capability that enables organizations to continuously develop and leverage digital capability.

#### 4.1.4 Digital technology capability

Digital technology capability refers to an organization’s ability to acquire, manage, integrate, and leverage digital technologies to create value for customers, suppliers, and the organization itself (DeLone et al., 2018; Proksch et al., 2024). This capability reflects a firm’s capacity to manage digital resources in ways that support business strategies, improve operational efficiency, and strengthen organizational competitiveness. According to Lu and Ramamurthy (Lu and Ramamurthy, 2011), digital technology capability involves the ability to acquire, share, integrate, and reconfigure digital resources in response to organizational needs and changes in the business environment. Therefore, its primary focus extends beyond technology ownership to encompass the organization’s ability to effectively manage and utilize digital technologies.

Some studies have focused on the adoption of specific technologies (Khin and Ho, 2019) or their implementation patterns (Elia et al., 2021), providing limited attention to the capabilities that enable such technologies to generate value. However, value creation from digital transformation largely depends on an organization’s ability to reconfigure its resources, including information technologies, artificial intelligence, machine learning, virtual reality, and various internet-based technologies (Heredia et al., 2022). This perspective suggests that technology becomes a source of competitive advantage only when supported by organizational capabilities that enable its integration and effective application within business processes.

The literature further indicates that digital technology capability is a critical driver of organizational innovation and competitive advantage. Renko et al. (2009) argue that information technology capability plays an important role in enhancing a firm’s innovation capacity. Similarly, Wiesböck et al. (2020) classify digital technology capability into three key components: a proactive orientation toward information technology, business capabilities that facilitate technology utilization (e.g., strategic partnerships), and technological infrastructure that serves as the foundation for digital transformation. Together, these components enable organizations to develop effective digital strategies through the digitalization of products and services (Denner et al., 2018) and the accelerated integration of customer feedback into product and service development processes (von Briel et al., 2018).

Digital technology capability also plays an important role in improving organizational performance. Ardolino et al. (2018), Khin and Ho (2019), and Wiesböck et al. (2020) found that the utilization of digital technologies contributes to greater innovation and product transformation. In addition, Zhang et al. (2022) demonstrated that strong digital capabilities can help organizations reduce unethical behavior in supply chains by enhancing transparency and technology-enabled monitoring. Nevertheless, digital technologies do not automatically generate organizational value. Lu and Ramamurthy (2011) and David-West et al. (2020) emphasize that the benefits of technology can only be fully realized when supported by effective management practices, competent human resources, and complementary organizational capabilities. Therefore, digital technology capability should be understood as a combination of technological resources and the organizational ability to manage, integrate, and leverage them in creating value and sustaining competitive advantage.

#### 4.1.5 Digital culture

Digital culture refers to the values, norms, practices, and expectations that shape individual and collective behavior within technology-enabled environments (Deuze, 2006). Similar to organizational culture more broadly, digital culture plays a critical role in determining the success of digital transformation initiatives (Fitzgerald et al., 2014) because it reflects an organization’s readiness and willingness to adopt and leverage digital technologies (Blatz et al., 2018). Digital culture can be viewed as an organizational mechanism that influences how technologies are accepted, utilized, and integrated into everyday business processes. Although increasingly recognized as an important factor in digital transformation, research on digital culture remains relatively limited compared with other dimensions of digital capability.

Several studies suggest that digital culture contributes to successful digitalization by strengthening digital strategies and enhancing the utilization of information technology capabilities (Proksch et al., 2024). El Sawy et al. (2016) argue that digital culture promotes behavioral change, improves organizational adaptability, and fosters tolerance for failure, which is often necessary in digital experimentation processes. Furthermore, Duerr et al. (2018) found that digital culture supports organizational knowledge creation, creativity, and innovation. In the context of human resource management, digital culture also plays an important role in enhancing employees’ digital skills. This can be achieved through reward systems, incentives, and management practices that encourage continuous learning and the sustained use of digital technologies (Lewis et al., 2004; Khin and Ho, 2019). Conversely, Fitzgerald et al. (2014) demonstrate that organizational cultures rooted in conventional practices can constitute a major barrier to digital transformation. Therefore, the development of a strong digital culture is a critical factor in building and sustaining organizational digital capability.

#### 4.1.6 Disciplinary perspectives on digital capability

The literature also varies across disciplinary traditions. In information systems research, DC is commonly examined in relation to IT resources, platform integration, analytics, and system-enabled value creation (Tan et al., 2013; Levallet and Chan, 2018). In strategy and management studies, the construct is more often linked to competitive advantage, dynamic capabilities, and strategic adaptation in digitally turbulent environments (Khin and Ho, 2019; Gao et al., 2022; Rupeika-Apoga et al., 2022). In operations and supply chain research, DC is associated with process improvement, service transformation, and digital supply chain responsiveness (Ardolino et al., 2018; Mourtzis, 2020; Queiroz et al., 2021). In HR and organizational behavior-related work, attention shifts toward employee digital skills, behavioral adaptation, training, and supportive cultural conditions (Xu et al., 2019; Scuotto et al., 2021). In marketing and innovation-oriented studies, DC is often linked to customer value creation, digital business models, and innovation performance (Torres de Oliveira et al., 2020; Elia et al., 2021). Taken together, these perspectives are complementary rather than mutually exclusive, but they study different parts of the same phenomenon and therefore produce different definitions and emphases.

### 4.2 Antecedents and consequences of digital capability: a multilevel synthesis

**Figure 5:**
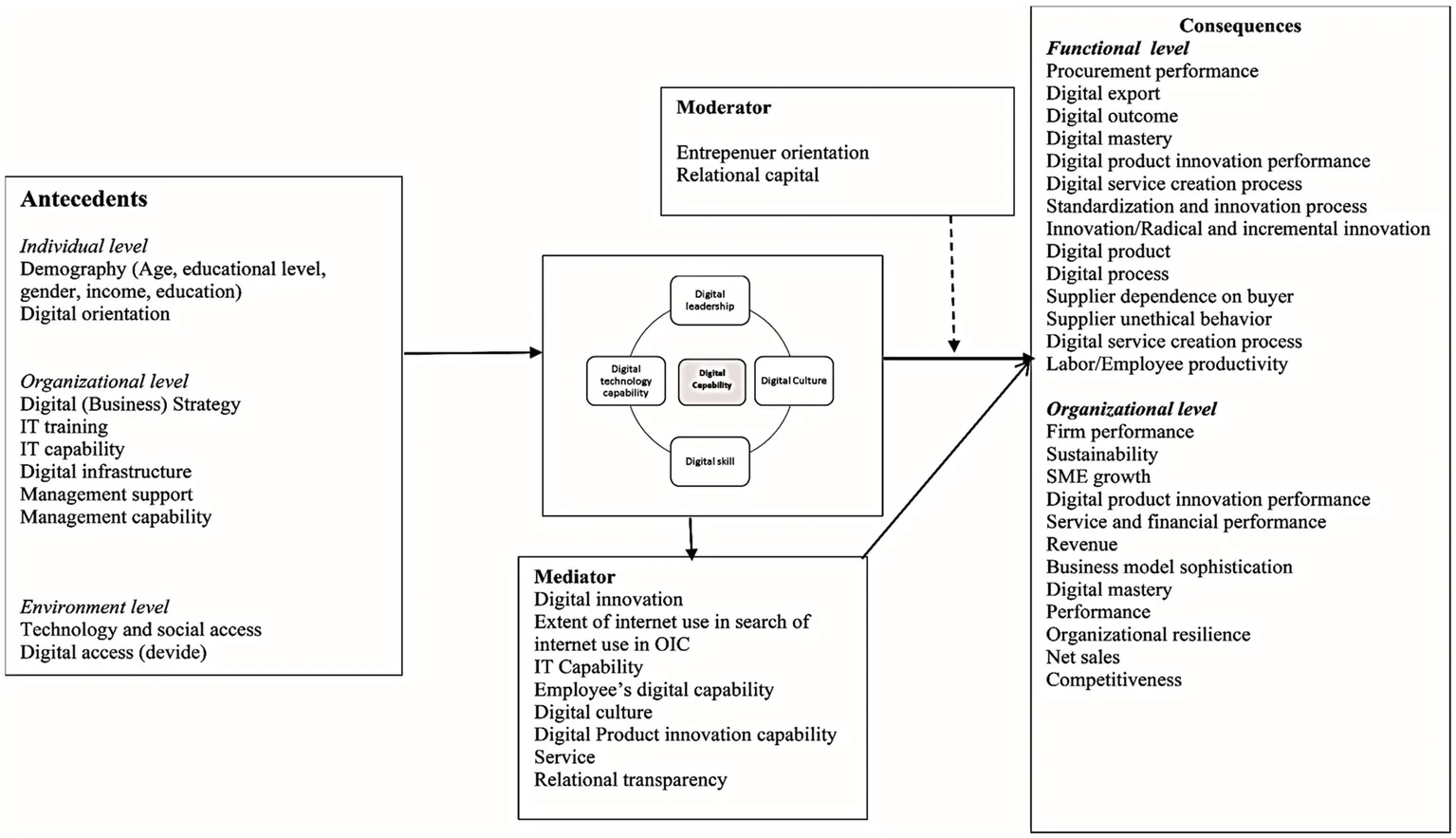
Antecedents and consequences of DC.

This section addresses the second review question (RQ2) by examining the antecedents, mechanisms, and consequences associated with DC in the reviewed studies. In addition to presenting the findings derived from the analyzed articles, including their methodological and theoretical frameworks, as well as conceptual definitions, this paper offers a synthesis of the empirical evidence pertaining to the factors that shape DC and their impact on performance. Furthermore, this study examines the mediating and moderating variables that influence the mechanisms and contexts through which DCs affect outcomes. These insights are drawn from a systematic literature review encompassing 27 empirical studies. Prior to the discussion, Figure 5 provides an overview of the results in this area.

#### 4.2.1 Antecedents

Based on the reviewed empirical studies, the antecedents of DC can be grouped into three broad levels: individual, organizational, and environmental. At the individual level, demographic and personal-enablement factors such as age, education, income, digital orientation, and self-efficacy are reported as relevant conditions shaping the development of DC (Wei et al., 2011; Hidalgo et al., 2020; Afrianty et al., 2022). At the organizational level, the literature points more consistently to digital strategy, IT training, infrastructure, and management support as enabling conditions that help translate individual potential into sustained organizational capability (Afrianty et al., 2022). At the environmental level, broader contextual conditions, particularly social access and internet accessibility, also appear to shape the opportunity structure within which DC develops (Chen and Wellman, 2003; Chang et al., 2015; Viard and Economides, 2015). Taken together, these findings suggest that DC does not emerge from a single source. It is shaped by the interaction of personal readiness, organizational support mechanisms, and the surrounding digital environment. However, the review also shows that genuinely multilevel empirical studies remain limited, which helps explain why the literature still lacks a fully integrated account of how these antecedents operate together.

#### 4.2.2 Consequences

The reviewed studies examined the consequences of DC mainly at operational and organizational levels. At the operational level, DC has been linked to outcomes such as procurement performance, digital product development, innovation-related outputs, and changing interorganizational dependence patterns (Torres de Oliveira et al., 2020; Wang et al., 2020; Son et al., 2021; Seclen-Luna et al., 2022). These studies show that the consequences of DC vary by functional domain. For example, in supply chain settings, capability asymmetry may increase supplier dependence rather than produce uniformly positive outcomes (Son et al., 2021), whereas in innovation-oriented settings DC is more often associated with improved digital product or market-facing outcomes (Torres de Oliveira et al., 2020).

At the organizational level, many studies report a positive association between DC and broader performance indicators such as financial performance, SME growth, resilience, or sustainability. However, the literature is not methodologically uniform enough to justify an unqualified claim of universal positive effects. A more accurate interpretation is that the reviewed studies generally point toward beneficial performance implications, while also indicating that the nature and strength of these effects depend on how DC is defined, which outcome is selected, and in what organizational context it is examined (Ray et al., 2004).

Taken together, the findings suggest that DC should be interpreted as a configurational construct rather than a single input–output factor. Its antecedents arise from multiple levels, and its consequences vary across domains and performance criteria. This helps explain why the literature appears mixed in emphasis even when many studies report broadly favorable outcomes. The review therefore supports a more cautious conclusion: DC is generally associated with advantageous organizational effects, but these effects are contingent on how the construct is composed, enacted, and measured.

#### 4.2.3 The mediating effect of DC

Six studies have investigated the mediation effect of DC on the outcome. Several mediators have been found to display organizational capabilities, including digital product innovation capability (Wiesböck et al., 2020) and IT capability (Proksch et al., 2024). As Andreeva and Ritala (2016) predict, DCs are high-level or generic capabilities that affect or update domain-specific or other capabilities. DC is also a mediator of several relationships between variables. In this study, DC mediates the effect of IT capability on employee productivity (Afrianty et al., 2022). In the context of the marketing field, DC becomes a mediator of digital strategy and digital products (Proksch et al., 2024).

#### 4.2.4 Moderator effect of DC

Few articles have examined the moderator of the relationship between DC and outcome. One study investigated the effect of entrepreneur orientation on the influence of digital marketing capability and firm performance (Wang et al., 2020). Another study employs relational capital on the impact of DC advantage on relational transparency between buyers and suppliers (Zhang et al., 2022). Furthermore, DC was found to moderate social media relationships with incremental and radical innovation (Torres de Oliveira et al., 2020).

Taken together, the reviewed studies suggest that DC is shaped by antecedents operating across multiple levels and produces consequences that vary by organizational context and outcome type. At the same time, the literature indicates that these relationships are not purely direct. Mediating and moderating factors help explain why DC may generate stronger effects in some settings than in others. This reinforces the need to interpret DC as a configurational and context-dependent capability rather than as a uniform predictor of performance.

### 4.3 Future research agenda

This section addresses the third review question (RQ3) by identifying promising avenues for future research based on the conceptual, empirical, and methodological gaps revealed in the reviewed studies. Rather than presenting isolated recommendations, the proposed agenda is organized around five interrelated priorities:

#### 4.3.1 Construct refinement and boundary clarification

One of the issues emerging from this review is the lack of conceptual consensus regarding the meaning and boundaries of digital capability (DC). While some studies conceptualize DC primarily as a technological capability (Ardolino et al., 2018; Wiesböck et al., 2020), others emphasize employee competencies (Scuotto et al., 2021), leadership capabilities (Nasution et al., 2020; Robertson et al., 2022), or broader organizational transformation capacities (Khin and Ho, 2019; Devi et al., 2022). Related concepts such as IT capability, digital orientation, digital maturity, digital competence, and digital innovation are often used interchangeably, resulting in conceptual ambiguity. Therefore, future research should focus on establishing clearer construct boundaries and validating whether the four dimensions identified in this review (digital skills, digital leadership, digital culture, and digital technology capability) constitute a stable higher-order construct across different organizational contexts. Researchers should also examine whether these dimensions are formative or reflective in nature and investigate whether alternative dimensional structures emerge across industries or at different stages of digital transformation. Future studies should more explicitly distinguish between the antecedents, dimensions, and outcomes of DC. For example, digital orientation is viewed as an antecedent by Khin and Ho (2019) and Rupeika-Apoga et al. (2022), whereas digital innovation is generally modeled as an outcome. Greater conceptual precision would contribute to cumulative theory development and enhance comparability across studies.

#### 4.3.2 Understanding digital capability as a multilevel phenomenon

This review suggests that digital capability (DC) is inherently a multilevel construct; however, the existing empirical evidence remains fragmented. Individual-level studies emphasize digital skills, digital literacy, and digital orientation (Wei et al., 2011; Hidalgo et al., 2020; Scuotto et al., 2021; Afrianty et al., 2022), whereas organizational-level studies focus on leadership, culture, technological infrastructure, and strategic alignment (Nasution et al., 2020; Devi et al., 2022; Proksch et al., 2024). Nevertheless, only a limited number of studies have simultaneously examined interactions across these levels. Future research should therefore investigate how individual capabilities are transformed into organizational capabilities through social, managerial, and structural processes. Questions such as how digital leadership shapes employee skill development, how digital culture facilitates capability accumulation, and how technological infrastructure enables capability deployment remain largely unanswered. Longitudinal multilevel research designs would be particularly valuable for understanding the emergence and evolution of digital capabilities over time. Our review also reveals evidence of capability gaps between the organizational and individual levels (Nasution et al., 2020), highlighting the need for further studies to explore capability alignment mechanisms and capability integration processes. Such research would provide a deeper understanding of how digital capabilities are developed, coordinated, and leveraged across different levels of analysis.

#### 4.3.3 Examining capability mechanisms, configurations, and boundary conditions

A substantial body of research has demonstrated a positive relationship between digital capability (DC) and organizational outcomes; however, the mechanisms through which these effects occur remain insufficiently understood. Several studies suggest that the effects of DC are mediated by digital innovation (Khin and Ho, 2019), digital transformation (2022), technological capability (Heredia et al., 2022), e-collaboration capability (Chi et al., 2018), relational transparency (Zhang et al., 2022), and organizational resilience (Robertson et al., 2022). These findings indicate that DC rarely produces outcomes directly. Accordingly, future research should move beyond direct-effect models and develop richer explanatory frameworks that incorporate both mediating and moderating mechanisms. Our review also reveals that the effects of DC vary across contexts. Factors such as entrepreneurial orientation (Wang et al., 2022), relational governance (Than et al., 2023), government support (Wahyuningtyas et al., 2021), firm size (Elia et al., 2021; Wang et al., 2022), and digital strategy (Proksch et al., 2024) appear to influence the outcomes of digital capability. Future studies should systematically investigate these boundary conditions and identify which combinations of capabilities are most effective under different environmental conditions. One particularly promising avenue is configurational research. Park and Mithas (2020) demonstrated that no single capability is sufficient to achieve superior performance; rather, performance emerges from nonlinear combinations of organizational capabilities. Future studies could employ fuzzy-set Qualitative Comparative Analysis (fsQCA) or other configurational approaches to examine alternative pathways through which DC contributes to organizational success.

#### 4.3.4 Exploring the dark side and relational consequences of digital capability

The existing literature predominantly emphasizes the positive consequences of digital capability (DC). However, several studies have revealed more complex and potentially negative outcomes. Son et al. (2021) found that digital capability asymmetry can increase SMEs’ dependence on more digitally advanced partners and expose them to opportunistic behavior. Mosch et al. (2022) demonstrated that service digitalization may alter power balances among firms, while Pagoropoulos et al. (2017) showed that capability development can enhance standardization while simultaneously constraining process innovation. These findings suggest that DC may generate unintended consequences, including dependency, power asymmetries, exclusion, capability inequality, and organizational rigidity. Future research should therefore investigate the dark side of DC and explore the conditions under which digital capabilities create vulnerabilities rather than advantages. In addition, greater attention should be devoted to interorganizational and ecosystem-level capabilities. Studies by Braun et al. (2025), Sun et al. (2021), and Galera-Zarco and Floros (2024) indicate that capability development increasingly occurs through partnerships, alliances, and digital ecosystems rather than solely within individual firms. Despite its growing importance, this remains an underdeveloped stream of research. Future studies should examine how digital capabilities are co-created, shared, and governed across organizational boundaries, as well as how ecosystem relationships influence capability development and organizational performance.

#### 4.3.5 Contextual and methodological expansion

The existing literature has largely focused on advanced economies, technology-intensive sectors, and large organizations. Although several recent studies have examined SMEs (Elia et al., 2021; Scuotto et al., 2021; Robertson et al., 2022; Proksch et al., 2024), evidence from emerging economies remains relatively limited and fragmented. Therefore, future research should extend its investigation to emerging economies, public organizations, cooperatives, family businesses, and resource-constrained SMEs. Findings from Heredia et al. (2022), Afrianty et al. (2022), Wahyuningtyas et al. (2021), and Ramantoko et al. (2018) suggest that capability development may follow different trajectories in these contexts due to variations in institutional support, digital infrastructure, and human capital availability. Methodologically, the field continues to be dominated by cross-sectional survey designs. Future researchers are encouraged to employ longitudinal approaches, mixed-methods research, and comparative case studies to capture the dynamic nature of capability development over time. Experimental and quasi-experimental designs may also provide stronger causal evidence regarding capability formation and its outcomes. Finally, future research could benefit from integrating complementary theoretical perspectives, such as Social Cognitive Theory, Resource Orchestration Theory, Institutional Theory, Ecosystem Theory, and Microfoundations Theory. Such integration would provide a richer understanding of how digital capabilities emerge, evolve, and generate value across different levels of analysis.

## 5 Limitations and future research directions

This study is subject to several limitations. First, it relies solely on the Scopus database, which may limit the comprehensiveness of the literature despite its broad coverage. Second, the application of strict inclusion criteria (restricting the sample to peer-reviewed journal articles in English within the business and management domain) may introduce publication and language bias. Third, the final sample of 53 studies, although rigorously selected, may constrain the generalizability of the findings in a rapidly evolving and multidisciplinary field. Fourth, the use of directed conceptual coding involves a degree of interpretive judgment, which may introduce subjectivity in the classification of antecedents and consequences. Fifth, this review did not assess inter-reviewer agreement through established reliability measures, such as Cohen’s kappa, which limits the ability to evaluate the consistency and reproducibility of the study selection and coding processes. Sixth, the review did not employ a standardized critical appraisal tool, such as the Critical Appraisal Skills Programme (CASP) or the Joanna Briggs Institute (JBI) appraisal framework, to systematically evaluate the methodological quality of the included studies. Consequently, the strength and robustness of the evidence underlying the review findings cannot be assessed comprehensively, and conclusions should therefore be interpreted with appropriate caution. Finally, the review is limited to the 2007–2022 period and reflects the fragmentation of existing literature, which remains uneven across levels of analysis, particularly with a stronger emphasis on individual-level perspectives.

Future research on digital capability (DC) should prioritize several key directions to advance theoretical and empirical development. First, greater construct clarity is needed through the empirical validation of its core dimensions (digital skills, digital leadership, digital culture, and digital technology capability) and the development of robust measurement instruments. Second, scholars should adopt multilevel perspectives to examine how individual, organizational, and environmental factors jointly shape DC, moving beyond fragmented single-level analyses. Third, future studies should further investigate the mechanisms and boundary conditions through which DC influences outcomes, including the roles of mediators, moderators, and contextual factors. Finally, expanding the contextual scope, particularly by focusing on SMEs and developing economies, will enhance the generalizability and practical relevance of findings. Methodologically, future reviews should incorporate formal inter-reviewer reliability assessments and employ established quality appraisal frameworks such as CASP or JBI to strengthen methodological rigor and improve the transparency of evidence evaluation. Longitudinal, multilevel, and mixed-method approaches are especially recommended, while theoretically, integrating perspectives such as dynamic capabilities, resource orchestration, and social cognitive theory can provide a more comprehensive understanding of how DC develops and operates across contexts.

## 6 Conclusion

This paper provides a systematic review of DC research and shows that the literature is more coherent than it first appears once its recurring dimensions and multilevel structure are made explicit. The review identifies four core dimensions (digital skills, digital leadership, digital culture, and digital technology capability) and argues that these should be understood as interdependent rather than isolated elements. It also shows that DC is shaped by antecedents at individual, organizational, and environmental levels, and that its consequences vary across operational and strategic domains rather than following a single universal performance pattern. By synthesizing these relationships into a multilevel framework, the paper moves beyond descriptive listing and offers a clearer conceptual basis for future empirical and theoretical work on DC.

The findings also carry implications for several stakeholder groups. For organizations, the review highlights the importance of treating DC as a coordinated capability rather than as a purely technological investment. For IT professionals and chief technology officers, the framework underscores the need to align digital infrastructure with leadership priorities, employee competences, and cultural readiness. For human resource practitioners, the findings emphasize the strategic role of training, digital skill development, and capability-building mechanisms. For business schools, trainers, and instructors involved in technological skill development, the review suggests that digital competence should be taught not only as a technical matter but also as part of broader organizational adaptation and value creation.

This paper presents a methodical review of digital capabilities (DC) literature and suggests directions for further study. By means of analysis of current research, we find areas to increase DC study. Our results highlight DC’s need as a fundamental organizational capacity for efficiently meeting market and technological needs. Though still a developing idea with different definitions and dimensions, DC has been the subject of prior research at both individual and organizational levels, including studies on mechanisms and contextual elements supporting its growth. To deepen knowledge of DC and direct future research, we underline the need of more empirical studies, methodological innovations, and enhanced theoretical frameworks.

## Data Availability

The raw data supporting the conclusions of this article will be made available by the authors, without undue reservation.
